# Cellular Bioenergetics and AMPK and TORC1 Signalling in Blood Lymphoblasts Are Biomarkers of Clinical Status in FMR1 Premutation Carriers

**DOI:** 10.3389/fpsyt.2021.747268

**Published:** 2021-11-22

**Authors:** Danuta Z. Loesch, Bruce E. Kemp, Minh Q. Bui, Paul R. Fisher, Claire Y. Allan, Oana Sanislav, Kevin R. W. Ngoei, Anna Atkinson, Flora Tassone, Sarah J. Annesley, Elsdon Storey

**Affiliations:** ^1^School of Psychology and Public Health, La Trobe University, Bundoora, VA, Australia; ^2^Mary MacKillop Institute for Health Research, Australian Catholic University, Melbourne, VA, Australia; ^3^St. Vincent's Institute of Medical Research and Department of Medicine, University of Melbourne, Fitzroy, VA, Australia; ^4^Centre for Molecular, Environmental, Genetic and Analytic, Epidemiology, University of Melbourne, Parkville, VA, Australia; ^5^Department of Physiology Anatomy and Microbiology, La Trobe University, Bundoora, VA, Australia; ^6^Department of Biochemistry and Molecular Medicine, School of Medicine, University of California, Davis, Sacramento, CA, United States; ^7^Department of Biochemistry and Molecular Medicine M.I.N.D. Institute, University of California Davis Medical Center, Davis, Sacramento, CA, United States; ^8^Department of Medicine (Neuroscience), Monash University, Alfred Hospital Campus, Melbourne, VIC, Australia

**Keywords:** FMR1 premutation, Fragile X-associated tremor/ataxia syndrome (FXTAS), motor scores, SCL-90 scale, lymphoblasts' bioenergetics measures, AMPK, TORC1, cognitive measures

## Abstract

Fragile X Associated Tremor/Ataxia Syndrome (FXTAS) is a neurodegenerative disorder affecting carriers of premutation alleles (PM) of the X-linked FMR1 gene, which contain CGG repeat expansions of 55–200 range in a non-coding region. This late-onset disorder is characterised by the presence of tremor/ataxia and cognitive decline, associated with the white matter lesions throughout the brain, especially involving the middle cerebellar peduncles. Nearly half of older male and ~ 20% of female PM carriers develop FXTAS. While there is evidence for mitochondrial dysfunction in neural and some peripheral tissues from FXTAS patients (though less obvious in the non-FXTAS PM carriers), the results from peripheral blood mononuclear cells (PBMC) are still controversial. Motor, cognitive, and neuropsychiatric impairments were correlated with measures of mitochondrial and non-mitochondrial respiratory activity, AMPK, and TORC1 cellular stress-sensing protein kinases, and CGG repeat size, in a sample of adult FXTAS male and female carriers. Moreover, the levels of these cellular measures, all derived from Epstein- Barr virus (EBV)- transformed and easily accessible blood lymphoblasts, were compared between the FXTAS (*N* = 23) and non-FXTAS (*n* = 30) subgroups, and with baseline data from 33 healthy non-carriers. A significant hyperactivity of cellular bioenergetics components as compared with the baseline data, more marked in the non-FXTAS PMs, was negatively correlated with repeat numbers at the lower end of the CGG-PM distribution. Significant associations of these components with motor impairment measures, including tremor-ataxia and parkinsonism, and neuropsychiatric changes, were prevalent in the FXTAS subgroup. Moreover, a striking elevation of AMPK activity, and a decrease in TORC1 levels, especially in the non-FXTAS carriers, were related to the size of CGG expansion. The bioenergetics changes in blood lymphoblasts are biomarkers of the clinical status of FMR1 carriers. The relationship between these changes and neurological involvement in the affected carriers suggests that brain bioenergetic alterations are reflected in this peripheral tissue. A possible neuroprotective role of stress sensing kinase, AMPK, in PM carriers, should be addressed in future longitudinal studies. A decreased level of TORC1—the mechanistic target of the rapamycin complex, suggests a possible future approach to therapy in FXTAS.

## Introduction

Fragile X premutations, which are small expansions of CGG repeat ranging from 55 to 200 in the non-coding region of the Fragile X Mental Retardation 1 (FMR1) X-linked gene, are associated with the variety of abnormal conditions (1). The most severe premutation-associated disorder affecting carriers of the FMR1 premutation allele is the late onset progressive neurodegenerative condition termed Fragile X Associated Tremor/Ataxia Syndrome (FXTAS), affecting 40−50% of male carriers after the age of 55, and 8–16.5% female carriers in the same age group (2–4). The much lower risk of FXTAS in females than in males may be, at least partly, attributed to the protective role of the normal FMR1 allele on the second X chromosome (5), but the existence of other sex-limited protective factors has recently been postulated (6). The standard diagnostic (core) features of FXTAS require one or more of the following pathological changes: intention tremor; cerebellar ataxia; and white matter disease in the middle cerebellar peduncles (MCP sign) seen on magnetic resonance imaging (MRI) (7, 8); white matter disease in the splenium of the corpus callosum (9) has more recently been considered another core FXTAS feature. Additional changes contributing to the diagnosis include parkinsonism, cognitive decline (executive function and memory deficits) in the later stages of this condition, neuropathy (10, 11), and other MRI findings such as global brain atrophy and white matter disease (9, 12–14), especially in the basis pontis, as well as around the lateral ventricles and deep white matter of cerebral hemispheres. Typical FXTAS neuropathological changes are of widespread ubiquitin-positive intranuclear inclusions abundant in neurones and astrocytes (15), extending to autonomic nervous and neuroendocrine systems and myocardial cells (16–18).

One component of the nuclear inclusions is FMR1 mRNA (19), which has previously been found to be elevated in the blood of premutation carriers as a function of increased CGG repeat number (20). These findings have led to a hypothesised pathogenetic mechanism that involves a toxic gain-of-function of the expanded CGG-repeat mRNA, which arises through the adventitious binding/sequestration by the CGG repeat of one or more proteins, contributing to dysfunction and/or death of the cell (21, 22). An alternative model for FXTAS pathogenesis has been proposed, in which “toxic” peptides are generated by initiating translation at non-AUG codons located upstream of the expanded CGG-repeat element repeat-Associated non-ATG (RAN) translation. This process, which generates a poly-glycine peptide that is toxic to cells and was detected in both the intranuclear inclusions of subjects with FXTAS and in the inclusions of the Dutch premutation CGG-repeat mouse model (23, 24). However, most recent analysis of these inclusions in FXTAS post-mortem brains revealed that they are composed principally of ~200 proteins, with over half involved in RNA binding and/or protein turnover, whilst the allegedly toxic poly-glycine peptide was found at extremely low levels (25). The abundance of the inclusion-associated ubiquitin -and small ubiquitin-like modifier (SUMO)-based modifiers suggests that the inclusions have been formed as the results of increased protein loads and elevated oxidative stress leading to maladaptive autophagy (25). These and other postulated mechanisms, associated with CGG expansions within the premutation range, and leading to the severe neuropathological changes underlying FXTAS, have been reviewed in (26).

Not all individuals carrying the premutation (PM) alleles exhibit the clinical phenotype of FXTAS: nearly half of male carriers do not develop this condition, but some proportion of these non-FXTAS individuals may manifest either its isolated features such as kinetic tremor or cognitive decline, or have other health problems, such as fibromyalgia, seizures, migraine, anxiety/depression, or hypertension, apparently occurring at higher frequencies than in the general population (27–29). A minority of male PM carriers remain asymptomatic regardless of their age.

As with many other neurodegenerative diseases, mitochondrial hypofunction has been suggested to play a role in the cytopathology of FXTAS. Several authors have investigated the status of mitochondrial expression and function in a range of cultured cells from adult PM individuals, including fibroblasts (30–32), as well as post-mortem brains (30, 33) and choroid plexus (34), reporting reduced expression and function of mitochondrial proteins. Mitochondrial hypofunction has also been encountered in the younger carriers (35).

This hypofunction was also encountered in peripheral blood mononuclear cells (PBMC) from PM carriers (32). A more recent multifaceted study of mitochondrial metabolism provided new details relating to metabolic deficits and mitochondrial dysfunction in this tissue from affected and non-affected female premutation carriers (36). These data obtained from human subjects have been supported by evidence based on neural tissue in Knock-In mice premutation models (33, 37, 38).

However, results from EBV-transformed B- lymphocytes (hereafter termed lymphoblasts) derived from a small sample of male individuals carrying PM alleles (39, 40) showed that mitochondria in those lymphoblasts are hyperactive, with elevation of all mitochondrial respiratory complex activities. These elevations were much more pronounced in the clinically less affected non-FXTAS PM carriers. It is unclear as to whether these elevations might parallel similar early changes in neural cells, or the features seen in short lived, metabolically quiescent peripheral lymphocytes do not reflect more advanced brain pathological processes.

In order to gain better insight into this phenomenon, here we correlate the measures of mitochondrial and non-mitochondrial respiratory activity with the motor, cognitive, and neuropsychiatric impairments seen in the affected PM carriers, on the one hand, and with the size of the CGG expansion, on the other, in a larger and more diverse sample of male and female premutation carriers. Moreover, we include two of the key proteins involved in cellular energy homeostasis: 5′ adenosine monophosphate-activated protein kinase (AMPK), the major sensor of inadequate cellular energy and other stresses (41), and Target of Rapamycin Complex I (TORC1), which is regulated by signals from AMPK, amino acids and growth factors (42, 43). Mitochondrial biogenesis and activity are regulated both by AMPK and TORC1, so the mitochondrial hyperactivity we previously observed in lymphoblasts from a small sample of PM individuals (40) could occur in response to hyperactivity of AMPK, which was indeed found to be elevated in lymphoblasts from a small sample of PM individuals (40). The present results provide new evidence for the relevance of AMPK and mTORC1 signalling status in blood lymphoblasts to the size of CGG expansion regardless of the clinical status of the PM carriers- as well as for the relationship between mitochondrial hyperactivity in these cells and motor, cognitive, and neuropsychiatric impairments in Fragile X Associated Tremor/Ataxia.

## Materials and Methods

### Sample

There were 28 male and 5 female normal controls, and 38 male and 15 female PM participants. All PM participants were adults. Except for one Asian (Chinese) male, all participants were white Caucasians. The source of all male and the 5 female PM participants was a major research project continuing from 2012 at La Trobe University and supported by the National Institutes of Health, USA. This project's male and female participants were originally recruited through fragile X families' admissions to the Victorian Genetic Counselling Clinic of the Murdoch Institute, or referred from several neurology clinics associated with the University of Melbourne and Monash University; the minority (some residing in the other states) were self-referred by postings in the community through The Australian Fragile X Association. Sixteen PM carrier males from this cohort were already included in our earlier publication, where basic cellular metabolism parameters were correlated with white matter lesion burden (39), and a further 6 males were included in a study of the relationship between AMPK and clinical and genotypic measures (40). Thus, of the 38 PM males, 16 were previously unreported. The other source of the female cohort (10 individuals) was an earlier 2008–2010 project supported by a research grant from the National Health and Medical Research of Australia (NHMRC) to ES and DL. These females, who had originally been ascertained either through their Fragile X Syndrome (FXS) children diagnosed at the Genetic Counselling clinics in the states of Victoria and South Australia, or were identified through cascade testing, were incorporated in an earlier study of progression of motor dysfunction based on a larger sample of female PM carriers (6). All PM participants were originally classified as belonging to the “FXTAS” spectrum (“FXTAS”), asymptomatic (“Unaffected”), and “Other” categories [as in: (40)]; the latter category comprising individuals with isolated features occurring in FXTAS, such as fibromyalgia, dementia, isolated kinetic tremor, mild ataxia, anxiety/depression, autism. However, for the purpose of present analysis, 11 carriers in the “Unaffected” category and 19 carriers in the “Other” category were combined into a non- FXTAS group. The healthy control group included 33 participants (with 5 females) recruited with funding support (to PRF, DL, ES, SJA) from the Michael J Fox Foundation as part of a parallel study on Parkinson's disease (2015–2017). All participants signed informed consent for the present study according to protocols approved by the La Trobe University Human Research Ethics Committee (HEC01-85 and HEC15-058).

### Protocols

#### Neurological and Cognitive Measures

Three motor scales: the Unified Parkinson's Disease Rating Scale Part III-Motor (UPDRS-III) (44); the International Cooperative Ataxia Rating Scale (ICARS) (45); and the Clinical Rating Scale for Tremor (CRST) (46) were administered by two neurologists with experience of these scales.

General cognitive functioning was assessed using Addenbrooke's Cognitive Examination Test (ACE-III) (47). The Similarities and Matrix Reasoning subtests of the Wechsler Adult Intelligence Scale (Third Edition; WAIS-III) (48) provided the measures of verbal and non-verbal reasoning, respectively. WAIS-III Digit Span Backward was employed as measure of working memory (48). Executive functioning was also assessed using The Symbol Digit Modalities Test as a measure of information processing speed (49). The delayed recall and discrimination indices of the Hopkins Verbal Learning Test-Revised (HVLT-R) (50), were employed as measures of delayed recall and recognition memory, respectively.

#### Psychiatric Pathology Test Scores

The Symptom Checklist 90 Revised (SCL-90-R) (51)—a 90 item self-administered questionnaire, was chosen as it can efficiently provide information on a broad range of relevant psychological symptom clusters. Here we report a summary score providing a measure of overall psychological distress—the Global Severity Index (GSI), as well as two specific symptom domains selected a priori: Depression and Anxiety.

#### FMR1 Molecular Measures

CGG sizing was conducted in the Laboratory of Dr. Tassone at the MIND Institute, UC Davis. Genomic DNA was isolated from peripheral blood lymphocytes using standard methods (Purygene Kit; Gentra,Inc., Minneapolis, MN). For Southern blot analysis, 10 micrograms of isolated DNA were digested with EcoRI and NruI. Hybridization was performed using the specific FMR1 genomic dig-labelled StB12.3 probe as previously described (52). Genomic DNA was also amplified by PCR (53).

#### Lymphoblast Culture

Lymphoblastoid cell lines were created by EBV-mediated transformation of cells from the Peripheral Blood Mononuclear Cell (PBMC) layer at the interface of Ficoll-paque Plus (Sigma-Aldrich) gradients as previously described (54).

Lymphoblasts were cultured in T25 flasks in growth medium (Minimum Essential Medium α (Gibco, Life Technologies), supplemented with 10% foetal Bovine Serum (FBS) and 1% Penicillin/Streptomycin) and were cultured in a humidified 5% CO_2_ incubator at 37°C. Cells were seeded to at least 2 × 105 cells/mL, and fed every 3 days either by replacing one third of culture medium with fresh medium, or split in a 1:3 ratio of cell culture to fresh medium. All experiments were conducted within 15 passages of recovery from frozen storage. Confluent cultures were harvested and resuspended in 250 μL aliquots in Recovery™ Cell Culture Freezing Medium (Gibco, Life Technologies) and stored at-−80°C. Frozen cells were recovered by thawing at 37°C and seeded into growth medium. Lymphoblast cultures were harvested for experimentation by centrifugation at 500 × g for 5 min.

#### Mitochondrial Mass and Membrane Potential

Two mitochondrial dyes, Mitotracker Green and Mitotracker® Red CMXRos (ThermoFisher Scientific), were used to estimate mitochondrial mass and membrane potential, as described by Missailidis et al. (55). Measurements were made in duplicate for each cell line, averaged and normalised within every experiment to the values for our standard selected control cell line (C105).

#### Seahorse Respirometry

Seahorse respirometry was conducted using the Seahorse XFe24 Extracellular Flux Analyzer and Seahorse XF24 FluxPaks (Agilent Technologies). Oxygen consumption rates (OCR) were measured in lymphoblasts that had been cultured in 6 well culture plates (corning) in growth medium prior to experiments. Experiments were conducted as described previously using 8 × 105 cells/well for each lymphoblast cell line. The basal O_2_ consumption rate (basal OCR), the decrease in OCR after oligomycin addition [OCR attributable to adenosine triphosphate (ATP) synthesis], the residual OCR after uncoupling with CCCP and blockade of electron transport with rotenone and antimycin A (“non-mitochondrial” OCR) and the “proton leak” (difference between OCR after oligomycin treatment and the “non-mitochondrial” OCR) in pmol/min/well were determined. The results were averaged over 4 replicate wells per experiment and at least 3 independent experiments per cell line.

#### AMPK Activity

AMPK assays were performed as described by us previously (54). Lysates were prepared from confluent cell lines (~25 ml) grown in T75 flasks, harvested, lysed in lysis buffer supplemented with phosphatase inhibitors (50 mM Tris.HCl pH 7.4, 150 mM NaCl, 1 mM EDTA, 1 mM EGTA, 1% Triton X-100, 50 mM NaF, 5 mM sodium pyrophosphate) then snap-frozen in liquid nitrogen. Thawed lysates were cleared by centrifugation at 10,000 ×g for 5 min. Supernatant total protein concentrations were determined with the PierceTM BCA Protein Assay Kit (Thermo Fisher Scientific). To concentrate the AMPK protein, 1 mg of total supernatant protein was immunoprecipitated with rabbit polyclonal anti-AMPKα1 antibody α1-(339–358) (56) bound to equilibrated protein A-agarose beads. The beads were recovered and washed four times by centrifugation before being resuspended in 60 μl wash buffer (50 mM HEPES pH 7.4, 150 mM NaCl, 10% glycerol, 0.1% Tween-20). This was named the AMPK slurry. AMPK activity was assayed over 10 min at 30°C by adding 20 μl of the AMPK slurry to 15 μl buffer (5 mM MgCl_2_, 50 mM HEPES pH 7.4, 0.1% Tween-20, and 1 mM DTT) containing 100 μM SAMS synthetic peptide (NH_2_-HMRSAMSGLHLVKRR-COOH). Reactions were started by adding [γ-32P]-ATP (final concentration 200 μM) and stopped by spotting 21 μl onto P81 ion-exchange chromatography paper (Whatman, GE Healthcare). Liquid scintillation counting (Perkin Elmer) was used to measure the incorporation of 32P into the SAMS peptide. Duplicates were averaged and normalised against the average value from all the control cell lines, in each independent experiment.

#### TORC1 Activity—Phosphorylation State

TORC1 activity in ME/CFS lymphoblast lysates was measured using a time-resolved fluorescence resonance energy transfer (FRET)based multiwell plate assay based on the phosphorylation state of 4E-BP1, a major TORC1 substrate (Cisbio Bioassays). Lymphoblasts were plated in duplicate wells for each cell line in growth medium at 5 × 104 cells/well in a 384-well plate. Lysis buffer was added to each well as per the manufacturer's instructions and the plate mixed on an orbital shaker for 40 min at RT. Lysates from each sample were then transferred to a white-bottom, white-sided 384 well plate (Corning, New York, USA) including various controls according to the manufacturer's instructions. Freshly prepared antibody mix was then added to each well (anti-4E-BP1 antibody labelled with d2 acceptor, and anti-phospho-4E-BP1 antibody labelled with Eu3+-cryptate donor). The plate was incubated at RT for 2 h and scanned using a Clariostar plate reader (BMG, Ortenberg, Germany) by reading the fluorescence emission at two different wavelengths (665 and 620 nm). The ratio of the FRET signal from anti-phospho-4E-BP1 antibody to the donor fluorescence signal from anti-4E-BP1 antibody was measured.

#### Reactive Oxygen Species (ROS)

Intracellular ROS levels were measured using the Fluorometric Intracellular ROS Kit (MAK145-1KT, Sigma). Lymphoblasts were seeded in triplicate in 90 μL of Dulbecco's phosphate buffered saline (Sigma) at 1.25 × 105 cells/well into a 96 well black, clear flat bottom plate. Fresh reaction mixture was prepared according to the manufacturer's instructions, 100 μL added to duplicate wells for each cell line and 100ul of PBS added to the remaining well to use for background subtraction. A cell- free control well containing PBS and reaction mix was also included. The plate was incubated in darkness for 1 h at 37°C with 5% CO_2_. The fluorescence was then read on a Clariostar microplate reader (excitation = 520, emission = 605 nm). A control cell line (C105) was included in each experiment to allow internal normalisation to control for between- experiment variation.

#### ATP Steady State Levels

Steady state ATP levels were measured using luciferase ATP-driven luminescence as per the manufacturer's instructions using the ATP Determination Kit (Molecular Probes) as described previously (54). The signal was normalised against that from a control cell line C105 used in every experiment as an internal control.

### Statistical Analyses

Pairwise comparison between median of normal controls (individuals in the normal CGG repeated range) vs. FXTAS, controls vs. PM non-FXTAS and FXTAS vs. non-FXTAS for cellular bioenergetics markers were carried out using the non-parametric Mann-Whitney test. For those variables that were significantly associated with age and/or gender, the analyses were conducted using residuals from regression on age or age and gender. The relationship between each cellular bioenergetics marker (outcome) and CGG repeat size was assessed using robust regression, adjusted for age or age and gender whenever appropriate. Spearman's rank correlation was used to compute pairwise correlations between pairs of cellular bioenergetics markers, separately for controls and FXTAS.

Robust regression was used to assess the relationship between each motor score (outcome) and cellular bioenergetics marker (predictor) in the FXTAS sample, adjusted for age and gender whenever appropriate. The Bonferroni correction was used to adjust for multiple testing. All analyses were conducted using software STATA statistical software (version 16.0, StataCorp, College Station, Tex., USA).

## Results

### Cellular Respiration and Bioenergetic Status in PM Carriers Compared With Non-carrier Controls

The two genetic groups (33 normal controls and 53 FMR1 PM carriers) bore alleles of the CGG trinucleotide repeat in the FMR1 locus that were either in the normal size range (20–40, control group), or in the premutation range (55–199, PM group). For the purpose of this study, the PM carriers were classified into non-FXTAS (*N* = 30, including 12 females), and FXTAS (*n* = 23, including 3 females) subgroups, with individuals in the latter subgroup diagnosed on the basis of the revised clinical criteria (57). However, sample sizes differ for individual traits depending on the proportion of missing data.

**Figure 1:**
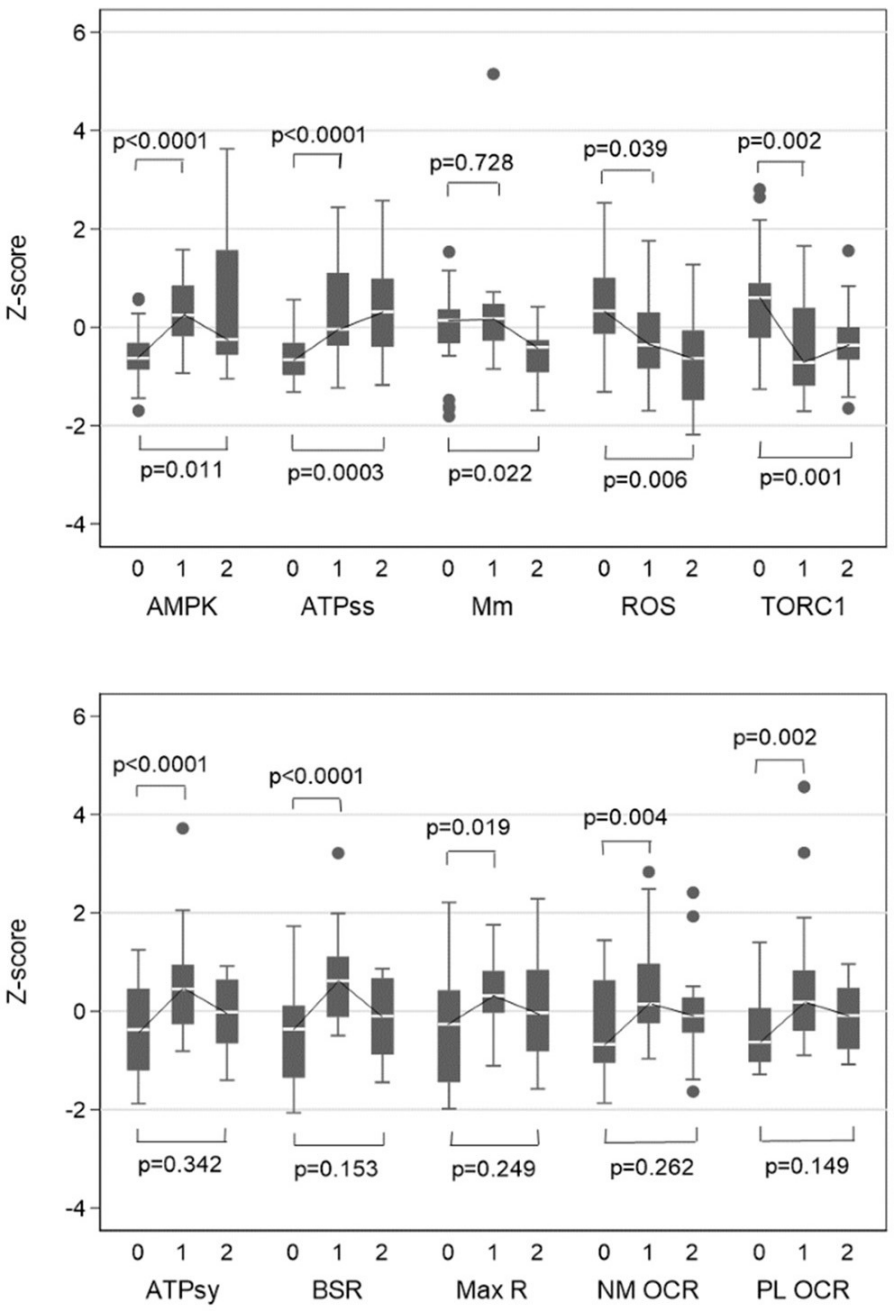
Box plots of Z-scores of biochemical variables for controls vs. Non-FXTAS and controls vs. FXTAS. 0, controls; 1, Non-FXTAS PM; 2, FXTAS; AMPK, Relative AMPK activity; ATPss, ATP steady state level; Mm, Mitochondrial mass; TORC1, TORC1 activity; ATPsy, ATP synthesis; BSR, Basal respiration rate; Max R, Maximum respiration; NM OCR, Non-mitochondrial ATP OCR; PL OCR, Proton Leak OCR. Z-score for BSR and TORC1 were adjusted for age, while ATPsy was adjusted for age and gender. Standardized measures (z-scores) for each variable were computed (for illustrative purpose only) using individual scores summed up over all three subgroups (data) according to the formula: z-score, (data-mean(data))/SD (data).

Since the main focus of this study has been on the relationships of cellular respiration and signalling measures (such as AMPK and TORC1) in blood lymphoblasts with motor and neuropsychiatric changes in the affected PM carriers, we first evaluated cellular functioning in the FXTAS subgroup, compared with non-FXTAS carriers and healthy controls. These results are summarised in Figure 1, for major components of cellular respiratory function (ROS) and cellular signalling (AMPK & TORC1 activities) (1a), and for mitochondrial and non-mitochondrial respiration parameters assessed by a Seahorse Respirometry tool (1b). The results show that the AMPK activity and ATP steady state levels are significantly elevated in both FXTAS and non-FXTAS subgroups compared with controls; while the levels of TORC1 activity and ROS levels are consistently reduced in both carrier subgroups compared with controls. In contrast with the above changes occurring in both carrier subgroups, mitochondrial mass is reduced only in the FXTAS subgroup.

Although the outcome of all Seahorse Respirometry measures are greatly increased in both non-FXTAS and FXTAS subgroups, this increase, relative to controls, is significant only in the former (1b). Direct comparison of these levels between the two subgroups shows, however, that the fall from non-FXTAS to FXTAS is significant only for Basal Respiration Rate (*p* = 0.0006) and ATP Synthesis (*p* = 0.0024).

**Figure 2:**
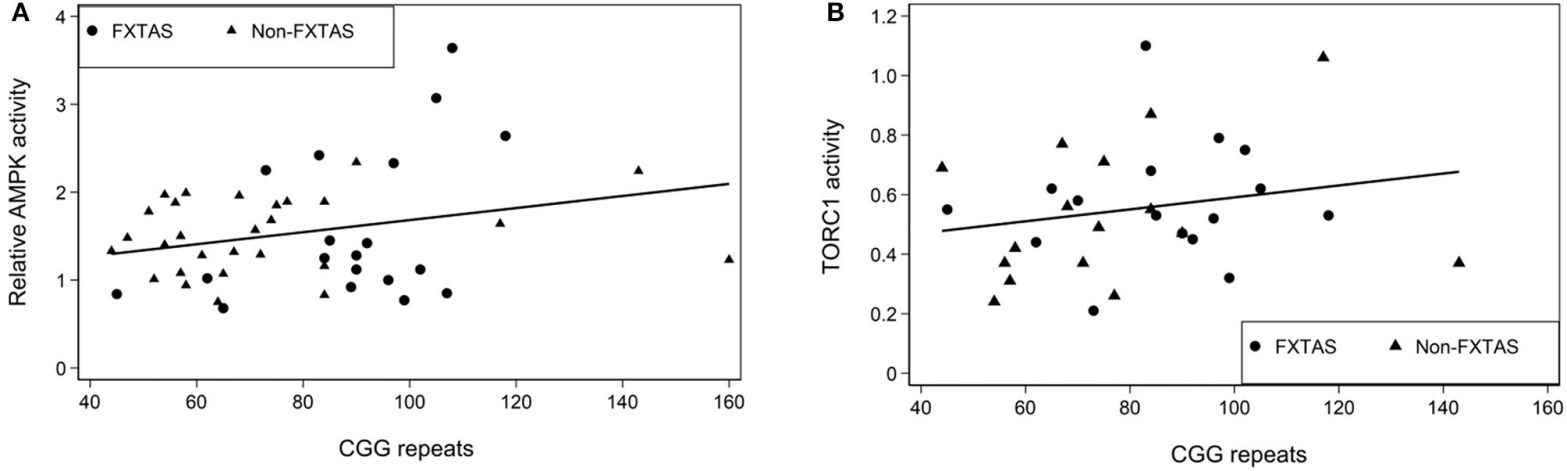
Scatterplots representing the linear relationship between relative AMPK activity **(A)** and TORC1 levels **(B)** in the total sample of PM carriers. Regression of AMPK on CGG: coefficient, 0.0052, *p* = 0.181; regression of TORC1 on CGG: coefficient, 0.0015, *p* = 0.404.

Consistent with this finding, several elevated Seahorse components—Basal Respiration Rate, ATP-Synthesis and ATP-Steady-State, and reduced level of ROS—all show significant (negative) correlations with CGG repeat size, exclusively in the non-FXTAS subgroup. There is also a positive correlation of CGG with mitochondrial mass in this subgroup. These results contrast with the FXTAS subgroup, where regression coefficients with the same cellular parameters have opposite sign, and the only significant relationship unique to this category occurred with Non-mitochondrial OCR (Table 1). Since CGG repeat expansion size is significantly (*p* < 0.0001) higher in FXTAS (89 median) compared with non-FXTAS (65 median) categories (insert in Table 1), both the sign and predominance of these correlations in the non-FXTAS group indicate that observed increases in the level of cellular bioenergetics, and decrease in the ROS levels, may be directly linked to repeat sizes within the lower end of CGG distribution for the premutation range. This may explain why, in this study sample, the Seahorse or other related components (except Basal Respiration Rate; *p* = 0.035) were not significantly related to CGG repeat size for the combined (FXTAS and non FXTAS) subgroups (data not shown). The same applies to the AMPK activity and TORC1 levels, though there is a trend, for both these components, to track the CGG repeat size within the < 100 range (see scatterplots in Figure 2).

**Table 1:** Relationship between biochemical variables (outcome) and CGG repeats, separately for FXTAS and Non-FXTAS assessed by robust regression.

	**FXTAS**				**Non-FXTAS**			
	**N**	**Coef**	**se**	*p* ^*^	**N**	**Coef**	**se**	p^*^
Relative AMPK activity+	19	1.85	0.96	0.054	28	0.30	0.33	0.364
TORC1 activity+	16	0.10	0.14	0.459	17	0.17	0.33	0.616
Mitochondrial mass+	10	−0.27	0.09	0.004^*^	15	0.10	0.03	0.001^*^
ROS+	10	0.26	0.82	0.753	15	−0.41	0.10	<0.001^*^
Basal respiration rate	17	3.00	1.72	0.081	26	−1.69	0.65	**0.009**
ATP Synthesis	17	1.32	1.36	0.332	26	−1.15	0.49	**0.019**
Non-mitochondrial ATP OCR	17	0.99	0.23	<0.001^*^	26	−0.28	0.20	0.169
Proton Leak OCR	17	0.46	0.36	0.195	26	−0.34	0.20	0.077
ATP steady state level+	18	0.84	1.01	0.409	27	−0.88	0.33	**0.008**
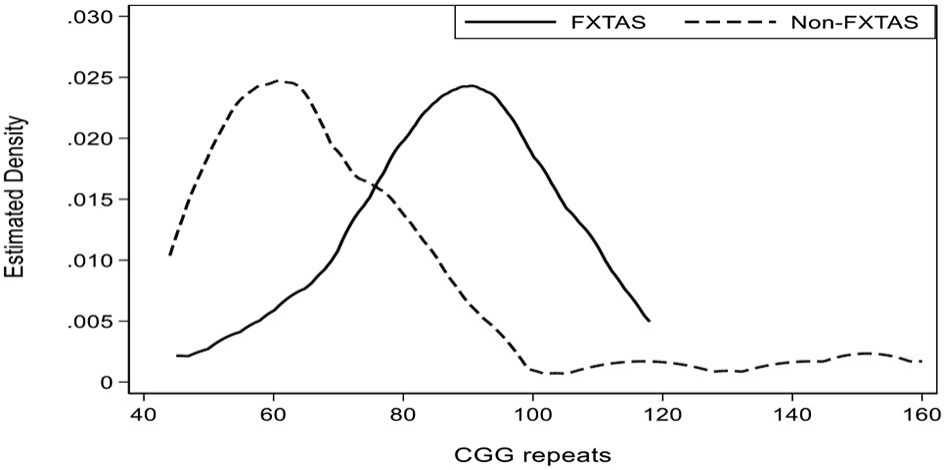			Kernel density estimate of distribution of CGG repeat numbers for FXTAS and non-FXTAS subgroups separately.					

*Analyses were conducted using robust linear regression; N, sample size; p, p-value; +Estimated regression coefficient (Coef) and standard error (se) were multiplied by 100*;

* *adjusted p-value remained < 0.05 after adjustment for multiple testing using Bonferroni correction method. Bold figures indicate significant relationships prior to adjustment for multiple testing*.

### Intercorrelations Between Cellular Markers in PM Carriers and Non-carrier Controls

Exploring intercorrelations between individual cellular bioenergetics components and major cellular energy sensors in PM carriers (especially in the FXTAS subgroup), and non-carrier healthy controls, may be helpful in the interpretation of their relationships with clinical changes. The results in Table 2 show that the cluster of four significantly intercorrelated major components of cellular bioenergetics—Basal Respiration Rate, ATP Synthesis, Non-mitochondrial OCR and Proton Leak—is identical in both FXTAS and control subgroups. Correlation coefficients are generally lower in FXTAS than in healthy controls, ranging from the highest of 0.925- between Basal respiration rate and ATP Synthesis (in controls), to the lowest of 0.463- between ATP Synthesis and Proton Leak (in FXTAS). Another important similarity between FXTAS and control subgroups is an absence of significant correlations between the above cluster of bioenergetics components, and AMPK, TORC1 and ROS, and between each other within this grouping. The only major difference between FXTAS and controls is the lack of significant relationship of Proton Leak with both Maximum Respiration and Non-mitochondrial OCR, and between the ATP synthesis and non-mitochondrial OCR, in the FXTAS subgroup; compared with the high correlations (0.70, 0.66, 0.63, respectively) seen in the controls (Table 2). Notably, in the non-FXTAS subgroup (data not shown), the only apparent difference with the controls is the lack of significant relationship between Proton Leak and ATP Synthesis; whereas the latter is, similarly to controls but in contrast with the FXTAS subgroup (shown in Table 2), significantly correlated with the non-mitochondrial OCR. Another potentially important difference between healthy control and FXTAS subgroups is the relatively high (0.54) negative correlation between AMPK and mitochondrial mass in FXTAS (although insignificant in a small sample of 10) compared with 0.027 -in a sample of 30 healthy controls (Table 2).

**Table 2:** Spearman's rank correlations among biochemical variables for controls (lower triangle) and FXTAS (upper triangle).

		**AMPK**	**BSR**	**ATPsy**	**NM OCR**	**PL OCR**	**ATPss**	**TORC1**	**ROS**	**Max R**	**Mm**
AMPK	Corr		0.31	0.39	0.10	−0.10	0.16	0.20	−0.07	0.17	−0.54
	N		19	19	19	19	20	18	9	19	10
	p		0.201	0.103	0.676	0.692	0.510	0.427	0.865	0.479	0.108
BSR	Corr	−0.06		**0.88**+	**0.58**	**0.64**	0.28	−0.16	0.42	**0.75**+	−0.13
	N	30		19	19	19	19	17	9	19	9
	p	0.769		**<**0.001	0.009	0.003	0.251	0.538	0.265	**<**0.001	0.732
ATPsy	Corr	−0.08	**0.93+**		0.29	**0.46**	0.26	−0.29	0.33	**0.51**	−0.03
	N	30	33		19	19	19	17	9	19	9
	p	0.664	**<**0.001		0.226	0.046	0.276	0.251	0.381	0.025	0.932
NM OCR	Corr	0.006	**0.82+**	**0.63**+		0.27	0.20	−0.08	−0.20	**0.72+**	−0.12
	N	30	33	33		19	19	17	9	19	9
	p	0.977	**<**0.001	**<**0.001		0.254	0.403	0.754	0.606	**<**0.001	0.765
PL OCR	Corr	−0.09	**0.72**+	**0.57**+	**0.66**+		0.04	0.24	0.28	0.44	−0.28
	N	29	32	32	32		19	17	9	19	9
	p	0.648	**<**0.001	0.001	**<**0.0001		0.870	0.358	0.460	0.063	0.460
ATPss	Corr	−0.17	−0.06	−0.05	0.06	−0.07		0.25	0.36	**0.46**	0.18
											
	N	30	33	33	33	32		19	10	19	9
	p	0.364	0.749	0.775	0.761	0.714		0.305	0.310	0.049	0.637
TORC1	Corr	−0.19	−0.26	−0.11	−0.35	−0.05	−0.31		−0.15	0.04	−0.57
	N	28	30	30	30	29	30		9	17	7
	p	0.333	0.158	0.579	0.057	0.785	0.095		0.700	0.881	0.180
ROS	Corr	0.12	0.09	0.17	−0.07	−0.27	−0.002	−0.12		0.07	0.19
	N	30	32	32	32	31	32	29		9	8
	p	0.530	0.644	0.357	0.691	0.141	0.993	0.547		0.865	0.651
Max R	Corr	−0.07	**0.89+**	**0.79+**	**0.81**+	**0.70**+	0.05	−0.27	0.11		0.02
	N	30	33	33	33	32	33	30	32		9
	p	0.696	**<**0.0001	0.0001	**<**0.001	**<**0.001	0.772	0.149	0.569		0.966
Mm	Corr	0.027	−0.09	−0.23	0.10	0.06	−0.04	−0.31	–**0.40**	−0.22	
	N	30	33	33	33	32	33	30	32	33	
	p	0.890	0.621	0.208	0.586	0.750	0.815	0.100	0.024	0.228	

*N = sample size; p = p-value; +adjusted p-value for Spearman's rank correlation (Corr) remained **<**0.05 after adjusting for multiple testing using Bonferroni correction. Bold figures indicate significant relationships prior to adjustment for multiple testing*.

### Relationships Between Cellular Bioenergetics Markers and Motor Dysfunction, Neuropsychiatric Changes and Cognitive Scores in FXTAS

The purpose of this aspect of our study has been to establish whether the changes in mitochondrial activity and cellular stress signalling encountered in blood lymphoblasts are significantly related to motor, neuropsychiatric and cognitive changes specifically occurring in clinically evident FXTAS. All individual differences between the increases in the three motor scores, and decreases in the cognitive scores included here, were highly significant between FXTAS and the non-FXTAS subgroups (*p* < 0.01). The sole exception was in the SCL90 scores—including GSI total, Depression and Anxiety domains.

**Figure 3:**
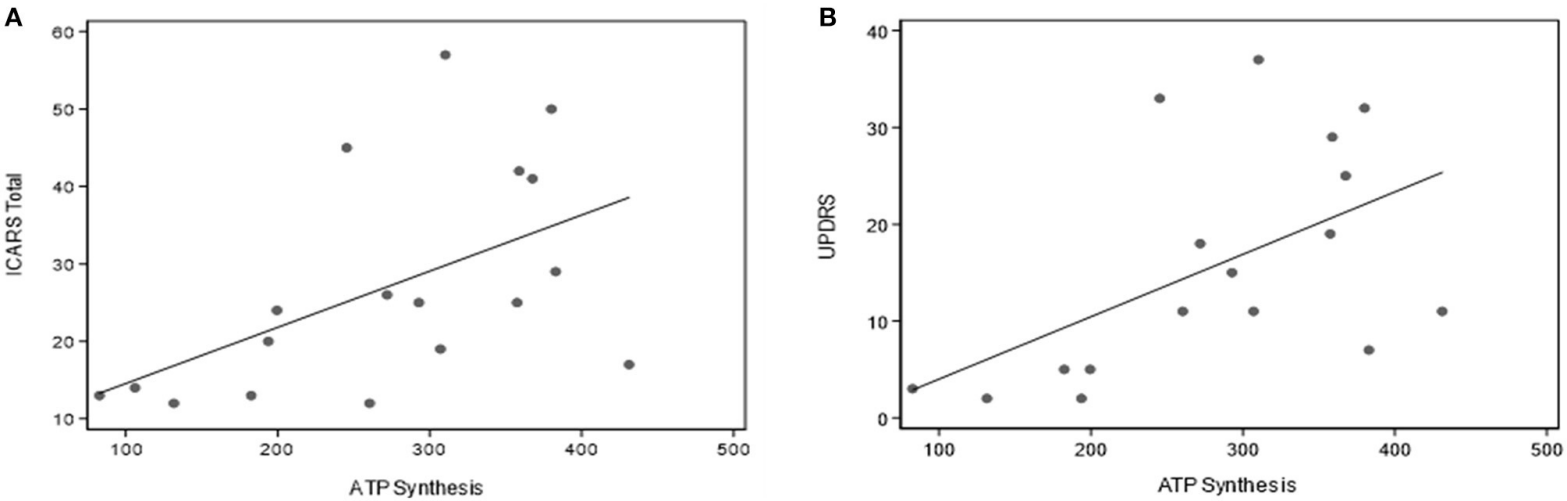
Scatterplots representing the linear relationships between ATP synthesis and ICARS **(A)** and UPDRS **(B)** scores in the total sample of PM carriers.

The data in Table 3A provides a summary of the relationships between key parameters of mitochondrial oxidative phosphorylation and motor impairments assessed by the three motor scales: ICARS, UPDRS, and Clinical Tremor. Notably, there are highly significant correlations between Basal Respiration Rate, Proton Leak and ATP synthesis, and each of the three motor scores (including Gait and Kinetic domains of ICARS). No other significant correlations survived Bonferroni correction. No such relationships have been encountered for the Non-mitochondrial component of Basal respiration, ROS, TORC1, or AMPK activity. It is particularly interesting to note that the score for parkinsonism (UPDRS), which is not a major feature of FXTAS as per standard clinical assessment, shows similar strong relationships with Seahorse major components as the scores for tremor/ataxia represented by ICARS (as illustrated in Figure 3 by scatterplots showing linear relationships of ATP synthesis with both ICARS total and UPDRS).

**Table 3A:** Relationships between motor scores and cellular stress sensing and bioenergetics measures in FXTAS sample.

	**Relative AMPK activity**				**TORC1 activity**				**Mitochondrial mass**			
	**N**	**Coef**	**se**	***p***	**N**	**Coef**	**se**	**p**	**N**	**Coef**	**se**	***p***
ICARS total	21	−0.20	2.86	0.945	18	3.09	12.4	0.803	9	−5.28	22.6	0.815
ICARS gait+	21	0.23	0.93	0.801	18	−0.65	4.40	0.883	9	−6.97	5.49	0.204
ICARS kinetic	21	0.21	1.59	0.897	18	3.11	5.81	0.592	9	−11.1	20.0	0.579
UPDRS+	19	0.87	2.55	0.733	17	−9.77	11.5	0.394	8	−21.8	26.8	0.416
TREMOR score	21	−1.56	3.34	0.642	18	−10.1	13.7	0.462	9	−21.1	48.2	0.662

	**ROS**				**Basal respiration rate+**				**ATP Synthesis+**			

ICARS total	9	−11.6	10.6	0.273	18	6.45	1.72	<0.001*	18	7.59	2.63	0.004*
ICARS gait+	9	−2.20	2.25	0.328	18	1.92	0.44	<0.001*	18	2.73	0.51	<0.001*
ICARS kinetic	9	−3.48	3.78	0.358	18	3.59	0.86	<0.001*	18	4.05	1.69	**0.016**
UPDRS+	8	−18.8	9.81	0.114	17	6.54	1.41	<0.001*	17	9.81	1.58	<0.001*
TREMOR score	9	−4.89	8.03	0.542	18	8.42	3.24	0.010*	18	7.44	3.09	**0.016**

	**Non**-**mitochondrial ATP OCR+**				**Proton Leak OCR+**				**ATP steady state level**			

ICARS total	18	18.7	14.9	0.211	18	33.5	7.44	**<** 0.001*	19	4.02	4.88	0.409
ICARS Gait+	18	5.57	3.43	0.104	18	6.88	2.48	0.006*	19	1.84	0.84	**0.029**
ICARS kinetic	18	8.89	6.71	0.185	18	18.0	4.08	<0.001*	19	1.41	2.74	0.608
UPDRS+	17	8.07	7.12	0.257	17	26.9	6.66	<0.001*	18	0.45	3.71	0.902
TREMOR score	18	24.2	16.8	0.151	18	51.5	16.6	0.002*	19	3.05	5.81	0.600

N = sample size; p = p-value; +Estimated regression coefficients (Coef) and standard errors (se) were multiplied by 100; +adjusted for age and gender if appropriate; *adjusted p-value remained **<**0.05 after adjustment for multiple testing using Bonferroni correction method. Bold figures indicate significant relationships prior to adjustment for multiple testing. Motor scale scores are the outcome, and cellular measures are predictors in robust regression analysis.

Notably, the Non-mitochondrial component, as well as the Basal Respiration Rate, are associated with each of the SCL90 measures—GSI Total, as well as Anxiety and Depression scores—while ATP Synthesis is associated only with the SCL90 Anxiety score. The latter is also (negatively) correlated with ROS (Table 3B). The depression domain of SCL90 score (*p* = 0.01) is also highly (negatively) correlated with CGG size (data not shown).

**Table 3B:** Relationships between SCL90 scores and cellular stress sensing and bioenergetics measures in FXTAS sample.

	**Relative AMPK activity**				**TORC1 activity**				**Mitochondrial mass**			
	**N**	**Coef**	**se**	***p***	**N**	**Coef**	**se**	***p***	**N**	**Coef**	**se**	***p***
SCL90 depression T score	12	1.27	3.60	0.725	10	−9.48	28.3	0.738	5	−15.0	47.3	0.751
SCl90 anxiety T score	12	1.26	3.85	0.743	10	6.30	37.1	0.865	5	−17.9	14.9	0.229
SCL90 GSI T score	12	0.88	2.09	0.675	10	2.18	13.3	0.870	5	−23.7	59.3	0.690

	**ROS**				**Basal respiration rate+**				**ATP Synthesis+**			

SCL90 depression T score	5	−18.6	20.0	0.352	11	6.81	1.66	<0.001*	11	4.47	3.46	0.197
SCL90 anxiety T score	5	−41.8	11.6	<0.001*	11	4.41	0.67	<0.001*	11	5.44	0.84	<0.001*
SCL90 GSI T score	5	−21.8	14.6	0.134	11	4.62	0.99	<0.001*	11	3.26	2.92	0.264

	**Non-mitochondrial ATP OCR+**				**Proton Leak OCR+**				**ATP steady state level**			

SCL90 depression T score	11	28.7	5.03	<0.001*	11	20.4	13.2	0.121	12	3.68	5.15	0.475
SCL90 anxiety T score	11	11.0	3.09	**<** 0.001*	11	17.7	9.58	0.065	12	1.56	4.28	0.715
SCL90 GSI T score	11	15.6	1.81	<0.001*	11	16.8	9.15	0.066	12	1.79	3.09	0.563

N = sample size; p = p-value; +Estimated regression coefficient (Coef) and standard error (se) were multiple by 100. *p-value remained **<**0.05 after adjustment for multiple testing using Bonferroni correction. Bold figures indicate significant relationships prior to adjustment for multiple testing. SCL90 scale scores (not adjusted for age or gender) are the outcome, and cellular measures are predictors in robust regression analysis.

In contrast, no significant relationships were encountered between cellular stress sensing or bioenergetics biomarkers and any cognitive measures. However, ACE-III total, used as a measure of global cognition, and Matrix Reasoning (a measure of non-verbal reasoning), show significant correlations with CGG repeat size in the FXTAS subgroup (*p* = 0.008 and *p* < 0.001, respectively). Matrix Reasoning, and Digit Span Backwards -another measure of executive functioning /working memory—both known to be impaired in otherwise unaffected PM carriers, are also correlated with CGG repeat size in the non-FXTAS subgroup (with *p*-values of 0.041 and 0.004, respectively). We did not observe any relationship of any other cognitive assays, or motor scores, with either CGG repeat size, or any bioenergetics or cellular stress response markers in this subgroup.

## Discussion

Since the first report by Ross-Inta et al. (30), strong evidence has accumulated for decreased mitochondrial bioenergetics in adult FMR1 premutation carriers with or without diagnosable FXTAS. Further data has consistently revealed various aspects of mitochondrial dysfunction in several human tissues-brain as well as peripheral (31, 32, 34, 36, 58, 59). This is supported by evidence based on Knock-in mice premutation models (37, 38). However, evidence from the small number of studies based on blood mononuclear cells (PBMCs) was controversial, showing the presence (36, 60) or the lack of (31) this dysfunction.

We previously conducted studies based on immortalised lymphocytes (lymphoblasts) derived from male individuals carrying premutation alleles. We have reviewed and discussed the value of using lymphoblastoid cell lines for this kind of study in our earlier publication (61). In contrast with the majority of those historical findings in other tissues, we reported elevated but functionally normal activities of all mitochondrial respiratory complexes compared with the same data from healthy non-carrier controls (39). Although we used cultured and EBV- transformed lymphoblasts in this study for both premutation and control non-carriers' samples, it is important to note that none of these cultures were allowed to proceed through more than a handful of cell doublings before use in these experiments and they were not used beyond 15 passages.

The above study marked the first time that these cells had been used in correlation with the phenotypic status of PM carriers. The results, based on a small sample of the FXTAS and non-affected carriers combined, showed an elevation of the Basal Respiration Rate and its three components measured by the Seahorse instrument: ATP synthesis; Proton Leak; and Non-mitochondrial OCR. Considering the nature of these findings, an obvious next step was to explore their relevance to FMR1-related clinical phenotypes. Indeed, we found, in the same study, that these increases showed linear association with the extent of white matter lesions, both total and in brain areas suggestive of FMR1 pathology. These data provided the first evidence for the relevance of the bioenergetic changes observed in blood lymphoblastoid cells to the presence and degree of FMR1-associated neurodegeneration.

In the following study (40), based on the same tissue and from the same sample of PM male carriers, we reported heightened cellular stress responses, as manifested by an increase in AMPK kinase activity in both FXTAS and non-FXTAS carrier subgroups, analysed separately. Notably, this increase was highly significant in the non-FXTAS carriers but was less evident in the FXTAS group. Furthermore, this activity showed significant (negative) correlations with the ICARS ataxia score, and with the extent of total- as well as supratentorial- white matter lesions, across the FXTAS and non-FXTAS samples combined. These results demonstrated that peripheral cellular stress responses and signalling have, as was the case with cellular bioenergetics components, meaningful links with pathological processes in the brain of male PM carriers.

In order to obtain clearer insight into the mechanisms behind these novel, but still fragmentary, findings in small samples, in the current study we expanded the scope of clinical measures, including all three motor scores, and several cognitive and neuropsychiatric scores, to explore the relevance of the thus-defined phenotype to the two major aspects of cellular pathology: cellular bioenergetics; and cellular stress responses. We used cultured lymphoblasts from a larger sample of carriers. Although we strongly emphasised the concept of a continuum of clinical and neuropathological changes in all PM carriers (2, 6, 62), here we distinguish between these two subgroups (FXTAS and non-FXTAS), with the intention of exploring the trajectory of cellular changes evolving from non- FXTAS to diagnosable FXTAS-as indicative of the potentially much more informative longitudinal approach.

The major finding from the primary comparative analysis, where we tracked the levels of major cellular bioenergetic measures from healthy controls across non-FXTAS to FXTAS subgroups, was a highly significant elevation of all the Seahorse respirometry (bioenergetics) components compared with healthy controls in the non-FXTAS subgroup, and a subsequent fall, in the FXTAS subgroup, to levels closer to, and not significantly elevated above, those of the control subgroup. It is possible however that the FXTAS-control differences are still present, and that the lack of significance is the consequence of the smallness of our sample. All the four major components showing consistent elevation are normally highly intercorrelated, as confirmed by our data from the normal control sample. These relationships were largely mirrored in the FXTAS subgroup, with the exception of proton leak, which was less strongly correlated with the remaining major bioenergetics components in FXTAS compared with healthy controls. These findings are clearly in contrast to the finding of mitochondrial dysfunction in blood lymphocytes from PM females (36), although not with the absence of dysfunction in the same cells from FXTAS males and females combined, as reported by (31).

Our results also show that smaller CGG repeat sizes within the PM range have the most prominent effect on these bioenergetics changes. Thus, the majority of significant correlations between these changes and CGG repeat size occurs in the non-FXTAS subgroup, where CGG size peaks at ~60 repeats; whereas in the FXTAS subgroup it peaks at ~90 repeats. The negative correlations between the elevation of these components and the CGG repeat size further strengthen the argument that the overstimulating effect on mitochondrial respiration rate is linked to the lowest end of CGG distribution within the premutation range. In contrast, the increasing CGG repeat numbers at the higher end of the distribution, (corresponding to the FXTAS subgroup), showed significant correlation with a decrease in the mitochondrial mass, as well as with an increase in non-mitochondrial oxygen consumption.

These data provide confirmatory evidence for an absence of detectable mitochondrial dysfunction in blood lymphoblasts from PM carriers without diagnosable FXTAS, which is consistent with the unchanged mitochondrial mass compared with controls. However, the obvious drop in the elevated cellular bioenergetics relative to the non-FXTAS subgroup, combined with a decrease of mitochondrial mass proportional to the increasing CGG repeat size may suggest a progression towards such dysfunction in the FXTAS subgroup. This is apparently consistent with the lack of significant relationship between non-mitochondrial OCR measures and ATP synthesis rates—exclusively in the FXTAS subgroup, which indicates that this synthesis may not be regulated homeostatically in the affected individuals.

Possible mechanisms behind this observed trajectory of Seahorse bioenergetics measures were discussed in our earlier reports (39, 54), but the more fundamental interpretation of these findings, which clearly contradict the evidence of dysfunction in other tissues from PM carriers, is still open to speculation. The most likely explanation is that the observed increase in cellular bioenergetics represents an early stage of cellular pathology in the form of an elevated response to cellular stress, linked to a small CCG repeat expansion. This mitochondrial hyperactivity may have a damaging effect on mitochondrial function, subsequently leading to its decline (with loss of mitochondrial mass) in the later stages of this process. However, the mitochondrial dysfunction stage may not be fully reflected in the lymphoblastoid cells because of the latter's rapid turnover, in contrast with other tissues such as neurones or fibroblasts (40, 54). More specifically, hyperactivity observed here may be related to the employment of B cells that are selectively immortalised by EBV transformation. Since these cells are involved in the adaptive immune system, they are expected to respond vigorously to the inflammatory component associated with neurodegenerative processes. This well-established association is exemplified by the report by Martinez Cardeno et al. (63) of an increased number and elevated activation state of microglial cells in about half (7 of 13) of their post-mortem FXTAS brains, indicating a neuroinflammatory state. Along the same lines, lymphocyte recruitment, activation and infiltration of the brain tissue has been reported in other neurodegenerative conditions, such as Parkinson's disease and amyotrophic lateral sclerosis (64).

Further insight into this dilemma may be provided by our parallel results from the current study, concerning cellular stress response and signalling in the two PM subgroups, FXTAS and non-FXTAS, represented by the dynamics of the AMPK-mTORC1 pathway. AMPK is a major factor sensing and controlling the level of cellular bioenergetics, mainly through enhancing ATP synthesis, switching off ATP -consuming anabolic pathways and enhancing mitochondrial biogenesis and activity, and autophagy [see: Hardie (65) for extensive review]. There has been strong evidence for multiple potential sources of neuronal stress linked to the elevation of the expanded FMR1 mRNA levels, which leads to corruption/sequestration of specific proteins (26). This phenomenon, combined with transcriptional induction of several stress response genes, was also observed in peripheral fibroblasts from affected (66, 67), as well unaffected (32) human PM carriers.

An increase in AMPK activity across the whole (premutation) range of repeat sizes observed here in cultured lymphoblasts from PM carriers, may be the result of those cellular stresses. The elevation of AMPK activity is typically regarded as adaptive, such that the prominent increase in this activity in the non-FXTAS subgroup was interpreted as being protective against cellular damage leading to FXTAS (39). Although this interpretation was mainly intuitive and based on our genotype-phenotype relationships results, a possible mechanism underlying its protective role in this context might be suggested by findings relevant to histone acetylation. Notably, AMPK has been reported as playing a significant role in the regulation of histone acetylation/deacetylation- as well as being regulated itself by acetylation [i.e., Vancura et al. (68)]. This report may be linked to the earlier data based on both Drosophila premutation model and lymphoblast cell lines derived from two premutation patients with probable FXTAS, showing that histone deacetylases suppress CGG repeat-induced neurodegeneration in FXTAS *via* transcriptional silencing (69). However, the effect of the double-edged sword of elevation of AMPK activity in cellular bioenergetics has also been reported in: (70–72). Since the observed decrease of AMPK activity in the FXTAS subgroup relative to its increase in the non-FXTAS subgroup was only slight and insignificant, our data does not provide adequate information to contemplate this option, which should be addressed in future follow-up studies based on larger samples.

Although the general view holds that there is close signalling interplay between mammalian Target of Rapamycin Complex I (mTORC1) and AMPK, especially in maintaining a balance between the level of anabolic and catabolic processes to preserve cellular homeostasis (73), our findings failed to show significant correlation between these two factors. This result may be partially due to the low statistical power of small samples, especially considering that the role of AMPK and TORC1 in the regulation of homeostasis dynamics may involve separate pathways, with both independently linked to CGG repeat expansions within the PM range. A complexity and multitude of factors affecting TORC1 modulation, and the protective effect of its inhibition, have been discussed earlier (73–75). By the same token, the lack of significant correlations between the levels of these two stress-sensing proteins and the clinical, as well as bioenergetics, measures of FXTAS in our data are not unexpected, considering that their primary controlling role is in cellular homeostasis, and thus their effect on the phenotype may be indirect. Nevertheless, our results clearly demonstrate that the levels of activity of both AMPK and mTORC1 in blood lymphoblasts from FMR1 premutation carriers are significantly related to, and are thus biomarkers of, clinical and genetic status of the carriers. Our comparative results demonstrated an inverse pattern of TORC1 to that of AMPK activities, by showing a highly significant decrease in non-FXTAS relative to healthy controls, and only a slight trend upward in the FXTAS-compared with the non-FXTAS subgroup. However, both AMPK and TORC1 showed a trend towards tracking the CGG repeat size in these two subgroups combined, though, in our data, the respective associations were not statistically significant. More detailed interpretation of the AMPK-TORC1 interrelationship and its relevance to the increasing CGG repeat expansion in the total sample of PM carriers have been given in our earlier publication (76). While the mechanism by which premutation alleles inhibit TORC1 activity is unknown, there are several possibilities including gain of function RNA toxicity (associated with RNA-mediated protein sequestration by elevated FMR1 mRNA levels) and polypeptide toxicity (from accumulation of abnormal, toxic polyG- or polyA-containing RAN translation products). Both of these processes could result in dysregulation of the AMPK-TORC1 signalling axis which regulates mitochondrial biogenesis and activity in response to diverse cellular stresses.

Notably, the decreased level of TORC1 found in our non-FXTAS PM subgroup is consistent with earlier results from the “90R” (premutation) mouse model (77). This showed a protective effect resulting from TORC1's inhibition through increased autophagy, and thus elimination of diseased cells. The recent analysis of the contents of intranuclear inclusions in human FXTAS neurons and astrocytes provided new evidence that elevated oxidative stress and increased loads of protein aggregates lead to these inclusions' formation through impaired autophagy (25). This may implicate mTORC1 as a potential treatment target. Indeed, a beneficial effect of rapamycin (which acts as an allosteric inhibitor of mTORC1) in alleviating non-motor symptoms of parkinsonism, has been reported in a mouse model of non-FMR1 related parkinsonism (78).

The present study is the first to provide evidence for the relationship between the severity of FXTAS neurological phenotype and cellular bioenergetics markers in blood lymphoblasts. These relationships were also significant in the combined non-FXTAS and FXTAS subgroups, but this aspect of analysis was focused on the subgroup meeting the FXTAS diagnostic criteria. This is because, although the non-Fragile X subgroup was not entirely asymptomatic, isolated symptoms occurring in these carriers may not have necessarily been relevant to the FXTAS phenotype, especially in the absence of overt (and age—unrelated) white matter changes in the brain.

All three key parameters of mitochondrial oxidative phosphorylation were highly correlated with each of the three motor scale scores: ICARS, Clinical tremor and UPDRS, in the FXTAS sample. That these correlations also involve the latter scale is somewhat unexpected but noteworthy, since, unlike tremor and ataxia, parkinsonism is not listed as a major feature of FXTAS. This particular result is of considerable interest, considering our earlier finding of a strong relationship between UPDRS score and CGG repeat size in FXTAS (6); and, more recently, new evidence for an elevated risk of parkinsonism in a sibship carrying the premutation/grey zone alleles and affected with kinetic tremor (79). It appears, therefore, that there is more overlap than previously thought between pathological mechanisms underlying both FXTAS and Parkinson's disease such that the former is often misdiagnosed as the latter.

Notably, the Basal Respiration Rate, ATP synthesis and Non-mitochondrial bioenergetics components are also highly correlated with the SCL90 GSI total, and /or Anxiety and Depression domains. This finding is not unexpected considering that neuropsychiatric features have been a major issue in both FXTAS and non-FXTAS carriers, especially females (6, 29, 80–83). Consistent with the extent of these problems across carrier categories, in this study we found significant correlations of these neuropsychiatric features with bioenergetic changes in the FXTAS subgroup, as well as in the combined (FXTAS and non-FXTAS) sample of PM carriers. This result calls for more attention to these well-documented and prevalent neuropsychiatric problems; since they occur both in obviously affected and non-affected PM carriers, the need for early intervention is emphasised, following our earlier recommendations for the female carriers (6). Moreover, both measures of executive functioning (Matrix Reasoning and Digit Span Backward), which are known to be affected early in the non-FXTAS carriers, are significantly correlated with the elevated bioenergetics and cellular stress response components in this subgroup. This suggests the relevance of the hyperactive energy metabolism observed in cultured lymphoblasts to early pathological processes in brain tissue underpinning those specific early clinical manifestations.

The absence of significant relationships between cellular stress sensing or bioenergetics biomarkers and any cognitive scores in the FXTAS subgroup is somewhat unexpected, with only two measures (ACE-III for global cognition, and Matrix Reasoning) being highly correlated with CGG repeat size in the same subgroup. The general absence of these relationships is in contrast with the earlier finding of significant correlation between bioenergetic markers and general cognitive measures, as well as selected measures of executive function (36). However, direct comparison cannot be drawn between the outcome of the two studies, since the earlier one was based on a different cell type (PBMCs), different measures of clinical and cognitive phenotype, and an overall different statistical approach. Apart from the small size of our samples, rigorous age adjustment, and correction for multiple testings, the reason for an absence of detectable relationships of cognitive impairments with our cellular biomarkers could be that cognitive decline is not a major or early feature of FXTAS. If indeed this decline relates to the later stages of the disease process, the relevance of this decline to the early changes represented in the short-lived lymphoblastoid cells (54) may not have been recorded. Clearly, further studies using a longitudinal model and a broader range of potential bioenergetics markers are required to fully address this issue.

In conclusion, one of the major findings from our study was the demonstration that the changes in bioenergetics and stress signalling occurring in cultured, EBV transformed lymphoblasts may be reliable biomarkers of motor and non-motor (especially neuropsychiatric) changes in PM carriers. A corollary of this would be that the dynamics of cellular changes in the transformed lymphoblasts should reflect the pathological processes in the brains of these carriers. An additional practical outcome of this result is that lymphoblastoid cell lines are a readily accessible and enduring cell type available from study participants.

Another major, though still not fully explained, finding is of hyperactivity of cellular bioenergetics components, especially in non-FXTAS PM carriers, compared with the lymphoblasts from non-carrier healthy controls. A particularly important aspect of these results is that the increased level of this hyperactivity in PM carriers is related to the CGG repeat size, but that this relationship is most apparent at the lower end of the CGG distribution.

Finally, the observed dynamics of the cellular stress-sensing protein kinases—AMPK and TORC1—raise an important issue of their possible role in protection against neural damage in FXTAS. These preliminary findings may guide future experimental work to establish the role of these energy sensors and metabolism-controlling enzymes, both being related to CGG repeat size, and both being potential targets for protective measures (such as rapamycin or metformin).

## Data Availability Statement

The raw data supporting the conclusions of this article will be made available by the authors, without undue reservation.

## Ethics Statement

The studies involving human participants were reviewed and approved by La Trobe University Human Research Ethics Committee (HEC01-85 and HEC15-058). The patients/participants provided their written informed consent to participate in this study.

## Author Contributions

DL: conception, organization, and partial execution of research project, neurological assessments and motor scales scoring, review of statistical analysis, and co-writing (with ES) a manuscript. BK: overseeing and coordinating stress response testing, interpretation of the tests result, contribution to study design, and reviewing and editing the final manuscript. MB: statistical analysis, contribution to study design, and to final editing of manuscript. PF: overseeing and coordinating cellular bioenergetics and stress response testing, interpretation of the test results, contribution to study design, and writing and editing the final manuscript. CA and OS: conducting cellular bioenergetics assays, interpretation of the results and entering and processing the data in the database file, and review of the manuscript. KN: conducting AMPK activity assays, interpretation of the results, review, and critique of the manuscript. AA: contribution to cognitive testing and scoring, creating study database, entering, and processing the data, contribution to statistical analysis, contribution to review, and final editing of manuscript. FT: conduct and interpretation of genetic molecular assays, review, and critique of manuscript. SA: coordination and conduction of cellular bioenergetics assays, interpretation of the tests result, contribution to study design, and writing and editing the final manuscript. ES: conception and partial execution of research project, neurological assessments and motor scales scoring, neuropsychological assessments or supervision of assessments, and co- writing (with DL) of manuscript. All authors contributed to the article and approved the submitted version.

## Funding

This study was supported by the National Institutes of Child Health and Human Development Grant, US, No HD 36071, to DL and FT, and by National Health and Medical Research Council Australia project Grant No CF06/0269 to ES, DL, and FT.

## Conflict of Interest

The authors declare that the research was conducted in the absence of any commercial or financial relationships that could be construed as a potential conflict of interest.

## Publisher's Note

All claims expressed in this article are solely those of the authors and do not necessarily represent those of their affiliated organizations, or those of the publisher, the editors and the reviewers. Any product that may be evaluated in this article, or claim that may be made by its manufacturer, is not guaranteed or endorsed by the publisher.
